# Social and Regional Disparities in Utilization of Maternal and Child Healthcare Services in India: A Study of the Post-National Health Mission Period

**DOI:** 10.3389/fped.2022.895033

**Published:** 2022-06-14

**Authors:** Madhumita Bango, Soumitra Ghosh

**Affiliations:** ^1^School of Health Systems Studies, Tata Institute of Social Sciences, Mumbai, India; ^2^Centre for Health, Policy, Planning, and Management, School of Health Systems Studies, Tata Institute of Social Sciences, Mumbai, India

**Keywords:** inequalities, maternal health, child health, healthcare utilization, NHM

## Abstract

**Background:**

India has enjoyed enhanced economic growth, but has fared poorly in human development indicators and health outcomes, over the last two decades. Significant health inequities and access to healthcare continue to exist and have widened within communities across states. This study examine the changes and disparities in maternal and child healthcare (MCH) among disadvantaged and advanced social groups in three states of India.

**Data and Methods:**

Four rounds of National Family Health Survey data were used to measure infant mortality rate (IMR) and under-five mortality rate (U5MR) according to the social groups for the selected states. This study investigates the socio-economic inequities manifested into caste and class differentials and inequities in availability, utilization, and affordability of maternal and healthcare services. Descriptive statistics and the logistic regression model were used. Individual- and household-level covariates were employed to understand the differentials in healthcare utilization.

**Results:**

The probability of not receiving full antenatal care (ANC) or full immunization for the children was highest among the Scheduled Caste/Scheduled Tribe (SC/ST) families, followed by economic class, mother's education and residence. Tamil Nadu showed the highest utilization of public health facilities, while Bihar was the poorest in terms of health outcomes and utilization of MCH care services even after the pre-National Health Mission (NHM) period. Bihar and West Bengal also showed private healthcare dependence.

**Conclusion:**

This study detected the presence of significant caste/tribe differentials in the utilization of MCH care services in the selected states of India. Limited accessibility and unavailability of complete healthcare were the foremost reasons for the under-utilization of these services, especially for people from disadvantaged social groups. The result also suggested that it is perilous to confirm “Health for All” immediately. It will be the efficiency with which India addresses inequities in providing healthcare services and guarantees quality care of health services.

## Background

India has enjoyed enhanced economic growth, but has fared poorly in human development indicators and health outcomes, over the last two decades. Health outcome indicators, such as child and maternal health, remain unsatisfactorily high compared with south and east Asian countries with similar income levels and economic growth rates. Aside from the low population level indicators, worrying inequities in health concur with the multiple axes of caste, class, gender, and geographical differences that have also been acknowledged (1–3).

Significant health inequities and access to healthcare continue to exist and have widened within communities and across states. In India, caste is an essential determinant of socio-economic inequities in nearly all wellbeing spheres. The constitution of India grouped various castes and tribes into four broad categories: Scheduled Castes (SCs), Scheduled Tribes (STs), Other Backward Classes (OBCs), and others. The SCs are castes that have historically faced social exclusions; they constitute about 16.6% of the Indian population, a large percentage of those live in rural areas and are mainly landless agricultural laborers. Like SCs, STs are a marginalized community and have been subjected to deprivation and discrimination for centuries and are also called Adivasis. As per Census 2011 figures, they comprised about 8.6% of India's population. OBCs and general castes together account for 71% of India's total population (4).

Several researchers found that various health outcome dissimilarities occur primarily from disparities in availability, accessibility, affordability, quality, and utilization of healthcare services (5, 6). Further, the inequalities in healthcare services lead to overall health disparities across regions and states of the population (6, 7).

Inequities in health are the unfair distribution of healthcare due to insufficient social arrangements and in India, this is a harsh reality. The recent round of the National Family Health Survey (8) data reveals sharp geographic and socio-economic divides in health outcomes. The deprived castes, the underprivileged and the less developed states excessively bear the burden of mortality. High rates of infant mortality (IMR) and under-five mortality (U5MR) and maternal mortality, in general, are inversely associated with income. These inequities are also accompanied by wide gaps across gender and caste (3, 9). However, reducing maternal and premature deaths by providing financial risk protection are key health-related targets of sustainable development goals (SDGs) (10). To achieve health-related SDGs, it requires substantial investment on maternal and child health (MHC) to protect households from high out-of-pocket payment shocks. High out-of-pocket expenditure (OOPE) is positively associated with limited access to healthcare, increases untreated morbidity, reduces consumption of goods and services, and leads to long-term disadvantage (11). The level of OOPE differs across socio-economic groups and type of health services (12–15).

## Maternal and Child Health in the Post-National Health Mission Period in India

**Figure 1:**
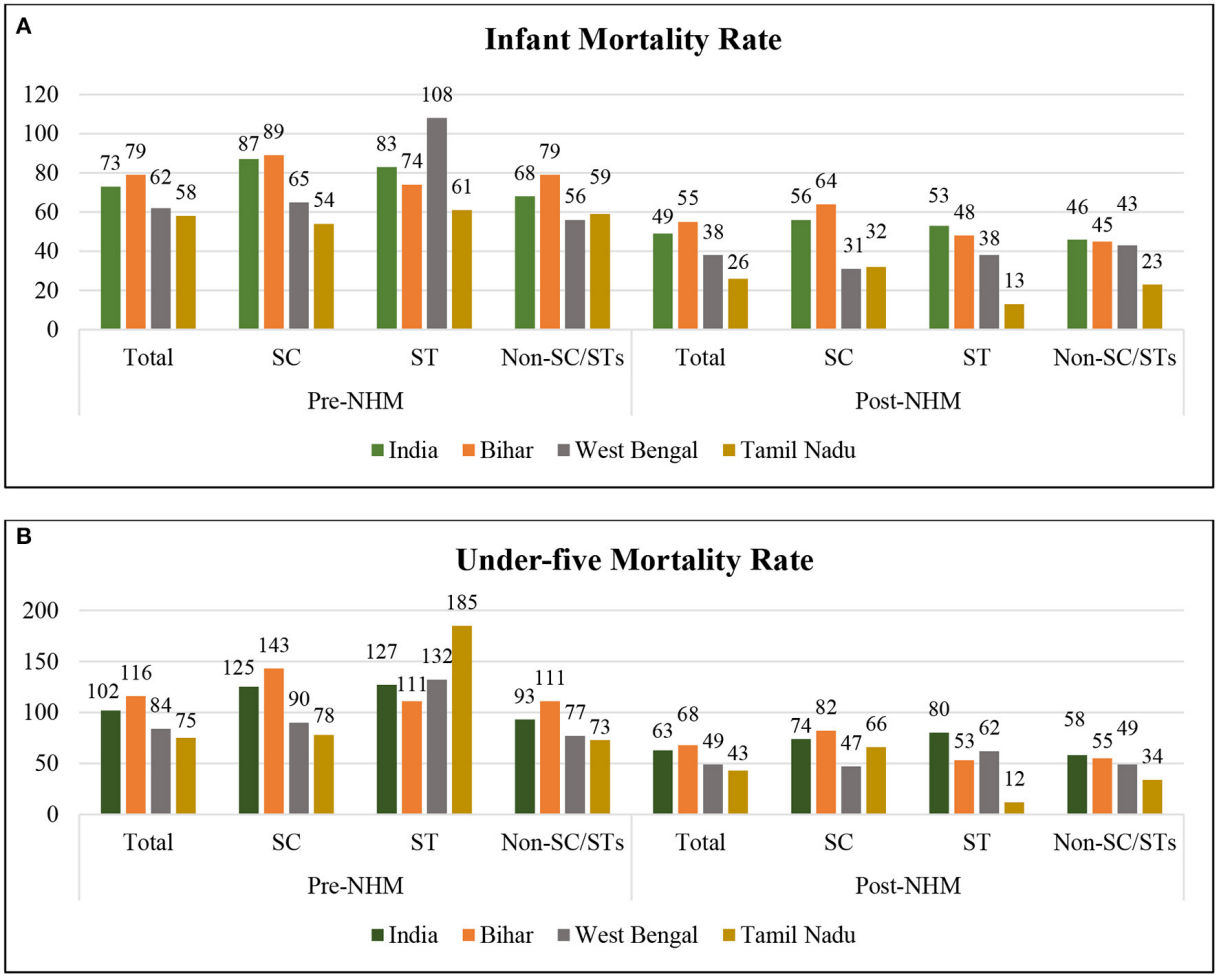
Social gap in **(A)** infant mortality rate (IMR) and **(B)** under-five mortality rate (U5MR) during the pre- and post-National Health Mission (NHM) periods. Authors estimated IMR and U5MR per 1,000 live births for the 5-year period preceding the survey. Average death rates have been calculated. For the pre-NHM period (1992–1999), NFHS 1 and 2, and for post-NHM period (2005–2016), NFHS 3 and 4 have been combined.

The healthcare system in India is categorized into two healthcare providers—public healthcare services, which are manifested in central, state, and local government, and private healthcare services. Fifteen years earlier in India, maternal and child deaths were very high with poor MCH indicators, especially in socially deprived groups. As a policy response, to reduce maternal and child mortality and reduce OOPE on MCH care, in 2005, the Government of India launched the National Rural Health Mission (NRHM) Program (16). The program intended to develop the health system by providing universal access to equitable, affordable, and quality maternal and child healthcare services. The NRHM was a wide-ranging, centrally driven government maternal and child health program that tried to address serious inequities such as under-investment, lack of human resources, infrastructure and some aspects of quality of care in the public healthcare sector (17–19). Initially, the NRHM was started in rural areas of 18 states, including 8 Empowered Action Group (EAG) states, with poor health infrastructure and the worst health indicators. Enjoying success in rural areas, the program was simultaneously started in urban areas and extended to non-EAG and other remaining states. Afterwards, the program was renamed as the National Health Mission (NHM). The two most critical apparatuses of the NHM are—Janani Suraksha Yojana (JSY) and Janani Shishu Suraksha Karyakaram (JSSK). The JSY is a cash enticement scheme provided to mothers for delivering at public health centers for the first or second time who are economically weaker (16). The incentive differed between rural and urban areas and state-to-state based on their performance and it covered the incentive to Accredited Social Health Activist (ASHA) workers too. The JSSK scheme was launched in 2011 and enabled all economically weaker pregnant women to deliver in public health facilities for no cost and free treatment for infants for a year (20). There has been significant progress in maternal and child health, after the implementation of the NHM in 2005. Trends in IMR and U5MR suggest (Figure 1) extensive improvements in the post-NHM period. Still, at the same time, inequalities in utilization of MCH care and health outcomes among the social groups have not been narrowed down.

Along with the child mortality rate, maternal mortality has also declined. JSY significantly impacted increasing natal care and JSSK has shown significant institutional delivery progression. The NHM proposed reducing the OOPE, besides improving maternal and child survival. The OOPE on delivery care largely diverges across states, classes and castes (21). Various studies explained that MCH care in India placed a high financial liability on households and it has also been recommended to reduce out-of-pocket payment to benefit disadvantaged groups (22–25).

However, some regions showed much progress in reducing maternal and child mortality and increasing access to MCH care. Nevertheless, progress has not been equitable and many women from deprived groups still lack access to maternal and child healthcare (26). This article aims to understand geographic and socio-economic inequalities in the selected states of India manifested in caste and class differentials and inequalities in the utilization, availability, and affordability of maternal and child health services in the post-NHM period.

## Methods

### Data

This study is based on the publicly available data from the fourth round of the National Family Health Survey (NFHS) conducted between 2015 and 2016. The NFHS 4 (8, 27) was conducted on behalf of the Ministry of Health and Family Welfare (MOHFW) and carried out by the International Institute for Population Sciences. This survey is an Indian version of the Demographic and Health Survey (DHS). This survey was conducted in over 29 states and 7 union territories (UTs). This survey was based on 1,315,617 births from 601,509 households with women aged 15–49 years old. The NFHS survey adopted a two-stage stratified random sampling approach by selecting primary sampling units (PSUs) and covering all 640 census enumeration blocks (CEBs) for urban areas and villages for rural areas with the probability proportional to population size (PPS) strategy. In the next stage, a systematic random sample selection of 22 households (HHs) was made for each PSU and CEB, respectively. Lastly, data from Central Bureau of Health Intelligence (CBHI) (28) 2016, Rural Health Statistics (RHS) (29) 2018–2019, and the Sample Registration System (SRS) maternal mortality ratio (MMR) Bulletin 2016–2018 (30) were also exploited.

### State Selection

We restricted our analysis to three states, namely, Bihar, West Bengal, and Tamil Nadu. Extreme health outcome variations and three kinds of health systems were identified between these three states. The third most populous state in India is Bihar, where around 40% of its population is below the poverty line. The state's significant health and demographic indicators, such as child mortality, MMR and birth rates, are much higher than the national level and replicate poor health outcomes. Bihar has been the lowest in the Human Development Index (HDI) for the last three. Substantial gaps in the health sector have also been found in infrastructure and essential health requirements (31).

On the other hand, Tamil Nadu is often ranked the best among the well-performing states in India, especially in public health delivery, next only to Kerala in terms of various health indicators and the HDI. The state is renowned for its low mortality rates, adequate healthcare infrastructure, and human health resources. Tamil Nadu has adopted various new approaches to improve access to good-quality healthcare services at an affordable cost. The public healthcare system in the state has enhanced over the last few decades, with more people having better access to medical care services, whereas in West Bengal, the healthcare system is a mix of public and private care, and the private healthcare system is flourishing at secondary and tertiary healthcare levels. To show inter-state differences, the said states were compared. The former had among the best and moderate indicators and the latter the worst indicators of health outcomes. In 2000, Jharkhand was removed from the southern portion of Bihar. For NFHS 1 and 2, the districts of the present Bihar state were combined to make it universal for the rest of the rounds (the districts under Jharkhand state were removed). For trend analysis, only the previous three rounds of NFHS data were utilized.

### Statistical Analysis

Descriptive statistics and the logistic regression model were used in these analyses. Individual- and household-level covariates were also utilized. The household characteristics pertained to the head of the household, while individual characteristics pertained to women (aged 15–49 years). The covariates were—age (15–24, 25–34, and 35–49 years), place of residence (urban, rural), caste (SC, ST, and non-SC/STs), religion (Hindu, Muslim, Christian, and others), wealth quantile (poor, middle, and rich), educational attainment (primary, secondary, and higher), husband's occupation (not working, professional or clerical, agricultural workers, and services), and place of delivery (public and private).

## Results

### Determining Factors of Health Inequalities

Among the SCs and STs, infant and under-five deaths were higher among the latter than the former. The averages for infant and under-five deaths decreased from 73 to 49 and 102 to 63, respectively, during the pre- to post-NHM period in India and all the selected states. However, this period was marked with increasing inequalities in child deaths among social groups, as shown in Figures 1A,B. Post-NHM, the IMR and U5MR showed a considerable decline in Tamil Nadu (23, 32) compared to other states. At the same time, India witnessed a tremendous social inequality in child deaths.

**Figure 2:**
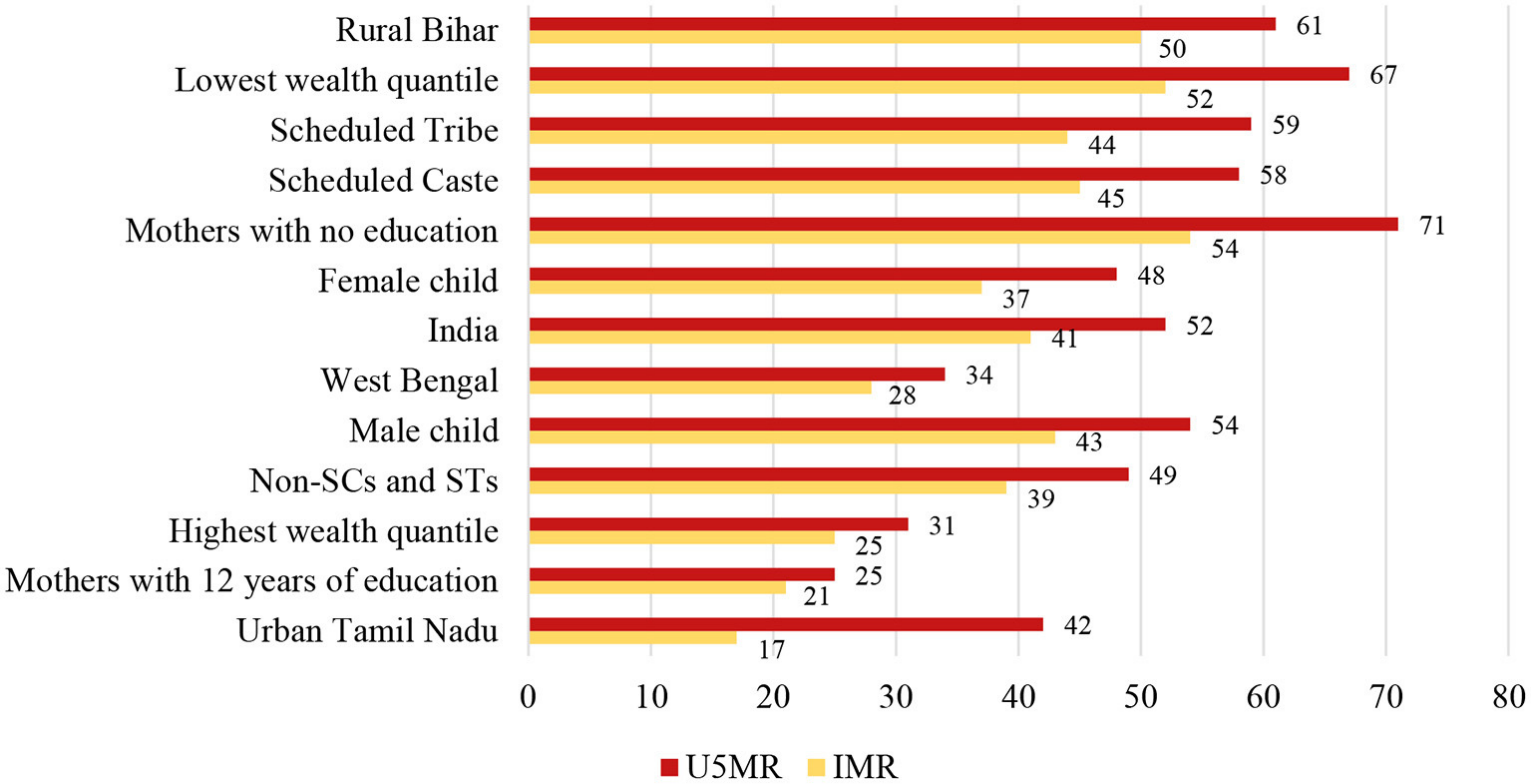
Inequalities in infant and under-five mortality in India, 2015–2016.

The recent round of NFHS data (2015–2016) acknowledged the sharp socio-economic and regional disparities in infant and under-five deaths and showed that those from deprived castes and poor and backward states bore the higher burden of mortality. However, the risks of mortality burden before reaching 1 or 5 years were higher among boys (43 and 54%, respectively) than girls (37 and 48%, respectively). Again, a persuasive mortality burden were shown to be higher among Scheduled Caste and Scheduled Tribe communities than non-SC/STs, followed by rural Bihar (the state with the poorest health indicators in India) than urban Tamil Nadu. However, mothers with 12 years of education had lower mortality among younger than 1- (21%) or 5-year-old (25%) children in India. Further, it can be explained from Figure 2 that economic status can be a significant indicator of infant and under-five deaths in India. Child deaths for the lowest income quantile earners were two times higher than that for the highest income quantile.

### Inequalities in Availability of Care Services

The availability of public health services is not even across the states of India (Table 1). Inequalities in public health infrastructure, resources (economic and human), and bed:population ratios have been recognized. Here, we compared Bihar, West Bengal, and Tamil Nadu based on public health infrastructure and availability of care services. Despite the rise in private healthcare facilities in Tamil Nadu, there were relatively better functioning primary public healthcare centers and better health outcomes in Tamil Nadu than Bihar and West Bengal or other states in India. This improvement might be due to the state government's investment in the health sector.

**Table 1:** Selected socio-economic and health indicators of India, Bihar, West Bengal, and Tamil Nadu.

**Indicators**	**India**	**Bihar**	**West Bengal**	**Tamil Nadu**
Population (in crores)	121	10	9	7
Sex Ratio (females per 1,000 males)	943	918	950	996
Per capita net state domestic product (Rs.)	86,879	36,143	78,903	128,366
Percentage of population below poverty line	22	34	20	11
Female literacy rate (%)	65	52	71	73
Average life expectancy (in years)	57	54	64	60
Infant mortality rate (per 1,000 live births)	41	48	28	20
Under-five mortality rate (per 1,000 live births)	52	59	34	45
Maternal mortality rate (per 100,000 live births)	113	149	98	60
Population covered by a sub-center	5,616	10,626	6,070	4,172
Population covered by a PHC	35,567	55,670	69,231	25,561
Population served per government hospital	64,425	70,701	138,792	58,697
Population per government hospital bed	1,678	8,789	1,341	1,170
Percentage of births delivered in a health facility	79	64	75	99
Percentage of deliveries attended by health professional	81	70	82	99
Percentage of mothers received JSY assistance after giving birth	43	57	30	31
Percentage of children (0–59 months) fully immunized	50	48	69	60

*National Family Health survey (NFHS-4) 2015-16; Central Bureau of Health Intelligence (CBHI) 2016; Rural Health Statistics (RHS) 2018-19; and SRS MMR Bulletin 2016-18*.

Along with the best health indicators, Tamil Nadu also had the best the socio-economic development in terms of—per capita income, sex ratio and female literacy. On the other hand, Bihar presented high poverty levels, poor health outcomes, inadequate public health facilities, utilization (population covered by a PHC, population:bed ratio, number of immunized children, and deliveries attended by a health professional), and poor social development. In comparison, West Bengal presented a mixed scenario of these two, with better use of public health facilities and availability of care services with improved social indicators.

### Inequalities in Utilization of Preventive and Curative Services

**Figure 3:**
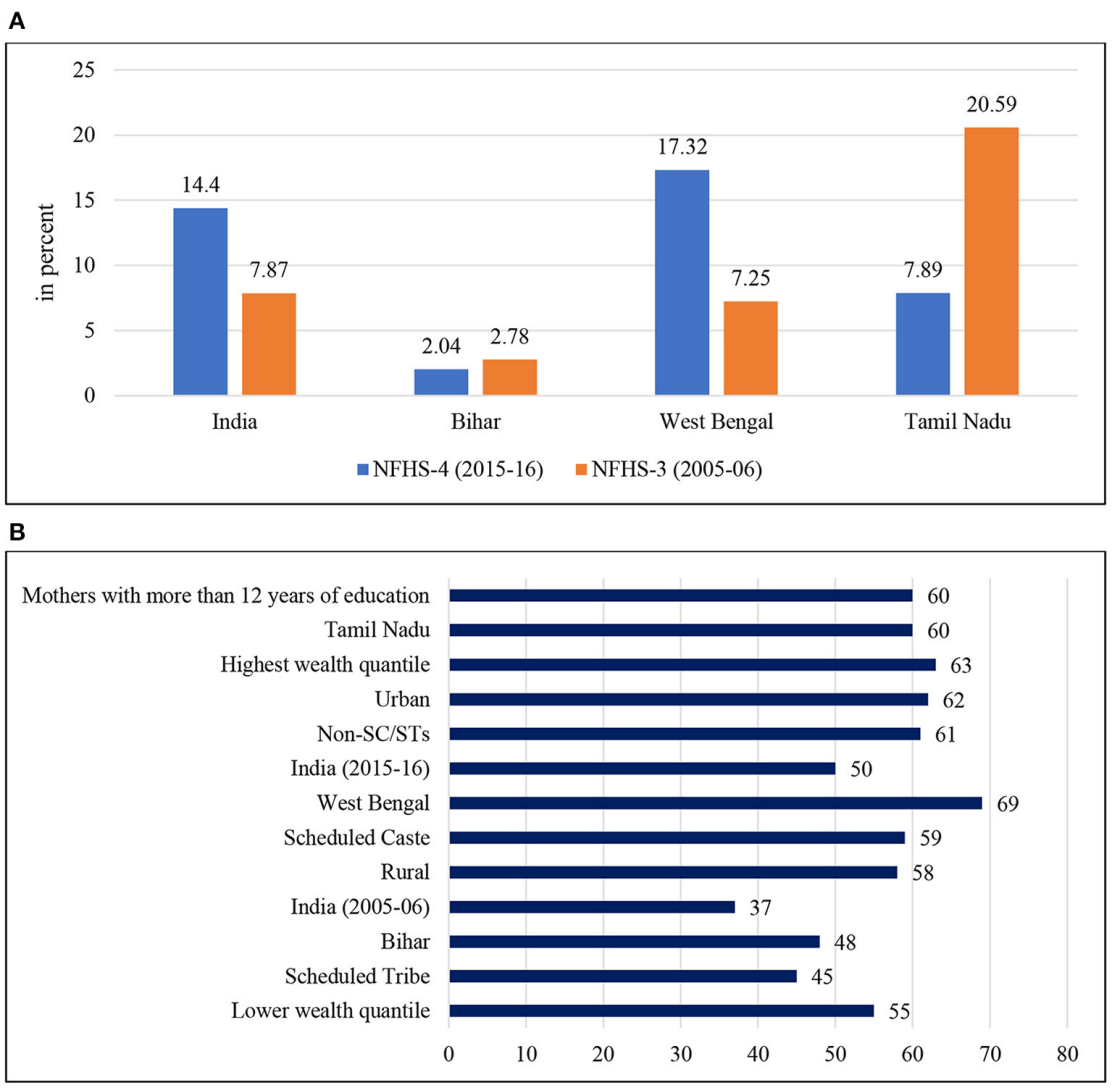
**(A)** Inequalities in receiving full antenatal care (women aged 15–49 years) in India. Full Antenatal Care comprises 4+ANC visits, 2+TT injections, and 100+IFA tablets. **(B)** Inequalities in immunization rate (full immunization of children aged 0–59 months) in India. Full immunization covers three injections each of DPT and Polio, and one injection each of BCG and Measles.

All India rates for receiving full antenatal care (ANC) increased to 14% in 2015–2016 from 8% in 2005–2006 (Figure 3), with an urban–rural differential of 23 and 11%, respectively. Unfortunately, for Tamil Nadu, a sharp 12% decline in full ANC coverage was reported, six percentage points less than the national level. In Bihar, the overall figure was lowest (2%) in 2015–2016, with a fall of 1% in 10 years, with a strident urban–rural difference of 4 and 2%. For West Bengal, a positive trend (with an increase of 10%) was recognized in full ANC coverage. Among the social caste categories (Table 2), the Scheduled Tribe population had the lowest percentage of receiving full ANC care in all the selected states. Again, there was considerable variation in the economic groups. The percentage of received ANC was highest among the richest quantile (25%) compared to the poorer section (7%) followed by mothers with a higher level of education (31%) in India. In contrast, data of full ANC coverage showed an upward trend in all the states, with some notable declines recognized among the top performers such as Tamil Nadu.

**Table 2:** Inequalities in receiving full antenatal care (women aged 15–49 years) by background characteristics in India, 2015–2016.

**Background characteristics**	**Full Antenatal Care**			
	**India**	**Bihar**	**West Bengal**	**Tamil Nadu**
**Place of residence**				
Urban	22.58	4.27	19.68	33.01
Rural	11.2	1.78	16.41	29.11
**Caste**				
Scheduled caste	12.85	0.94	18.67	29.27
Scheduled tribe	10.87	0.6	14.78	22.47
Non-SC/STs	15.48	2.44	18.28	31.85
**Religion**				
Hindu	14.45	2.1	19.25	30.84
Muslim	11.88	1.76	12.95	33.95
Christian	23.83	#	12.49	30.44
Others	20.9	#	11.16	#
**Wealth quantile**				
Poor	6.66	1.05	14.02	28.21
Middle	15.56	3.64	19.21	28.39
Rich	24.84	9.49	25.18	33.38
**Mother's educational attainment**				
No education	5.24	0.76	11.75	28.42
Primary	9.76	1.55	14.92	27.64
Secondary	18.06	3.44	18.16	29.75
Higher	30.99	12.82	34.18	36.02

*Full Antenatal Care comprises 4+ANC visits, 2+TT injections, and 100+IFA tablets*.

# *Not enough samples*.

The all-India average for full immunization coverage for 2005–2006 was 37% with an improvement of 50% for 2015–2016, with a four percentage point rural–urban difference. An intra-state variation was shown between Bihar, West Bengal, and Tamil Nadu. The overall full immunization coverage for Tamil Nadu was 60%, while the overall full immunization coverage for Bihar and West Bengal was 48 and 69%, respectively. A substantial percentage difference was recognized among the socio-economic groups. The coverage was lowest among SCs (59%) and STs (45%) compared to non-SC/STs (61%). In comparison, the coverage was highest in the wealthiest quantile (63%). However, the gap between the socio-economic groups has not been narrowed down even a decade after the NHM and Tamil Nadu showed a drop in full immunization coverage.

### Inequalities in Maternal and Child Health Expenditure Burden

**Figure 4:**
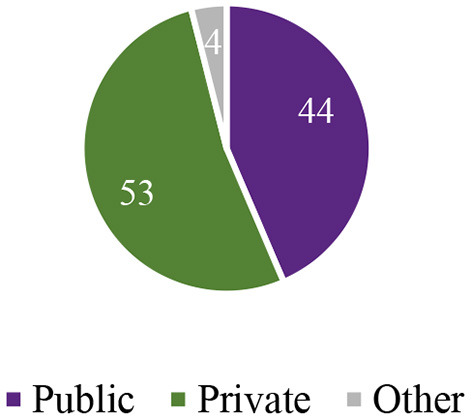
Percentage distribution of utilization of healthcare facilities in India, 2015–16.

The evidence (Figure 4) from recent years showed a dependence on the private sector (53%); this may be due to India's weak public health delivery systems, while rural dependency on the public sector has been recognized. Further, intra-state variations occurred in the utilization of healthcare services.

Tamil Nadu had better developed and administrated services at the primary level, showing a higher level of utilization of the public sector (77%) even more than the national level. In contrast, a poorer state such as Bihar showed a lower utilization of the public health sector (22%). In West Bengal, this percentage was mixed with more utilization of the public sector (55%).

**Figure 5:**
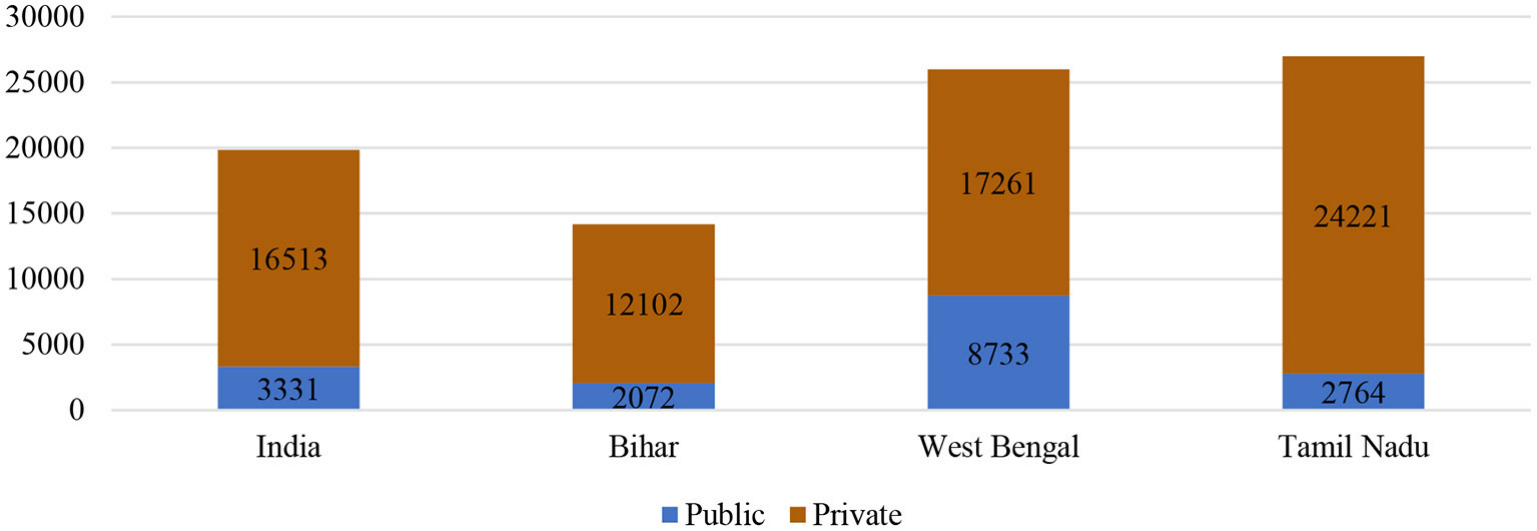
Burden of out-of-pocket expenditure (in rupees) for delivery of the most recent live birth among women aged 15–49 years in India and selected states, 2015–2016.

**Figure 6:**
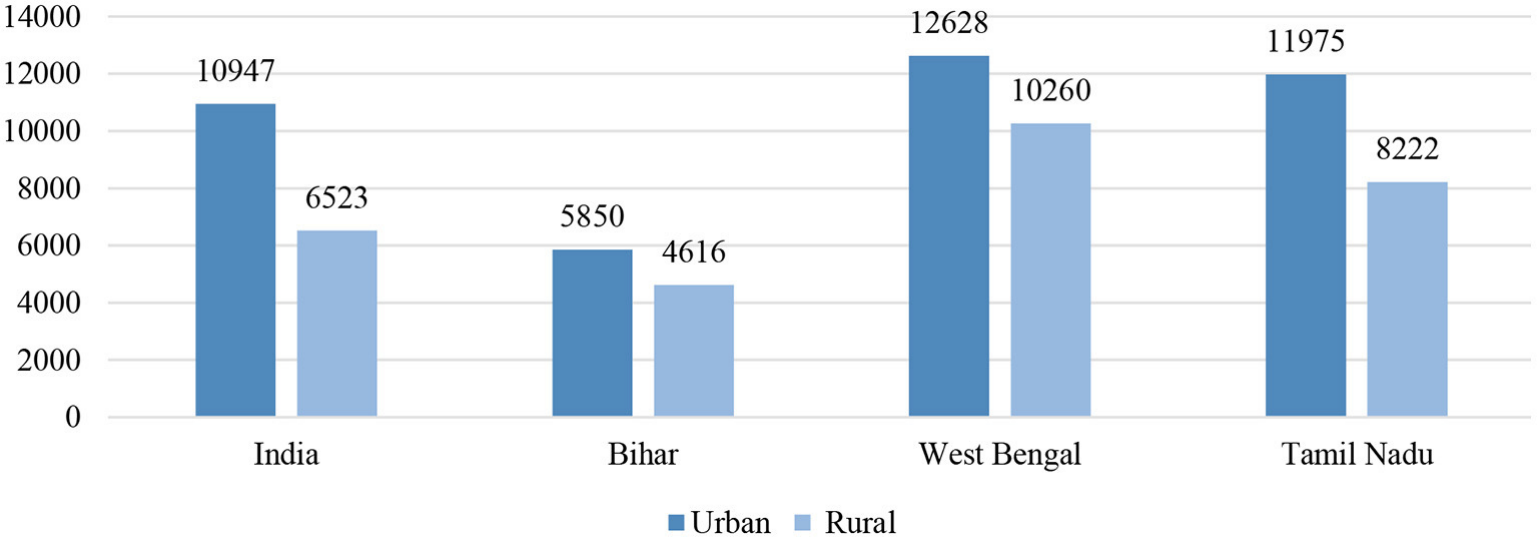
Urban–rural differences in average out-of-pocket cost (in rupees) paid for delivery of the most recent live birth among women aged 15–49 years in India and selected states, 2015–2016.

**Figure 7:**
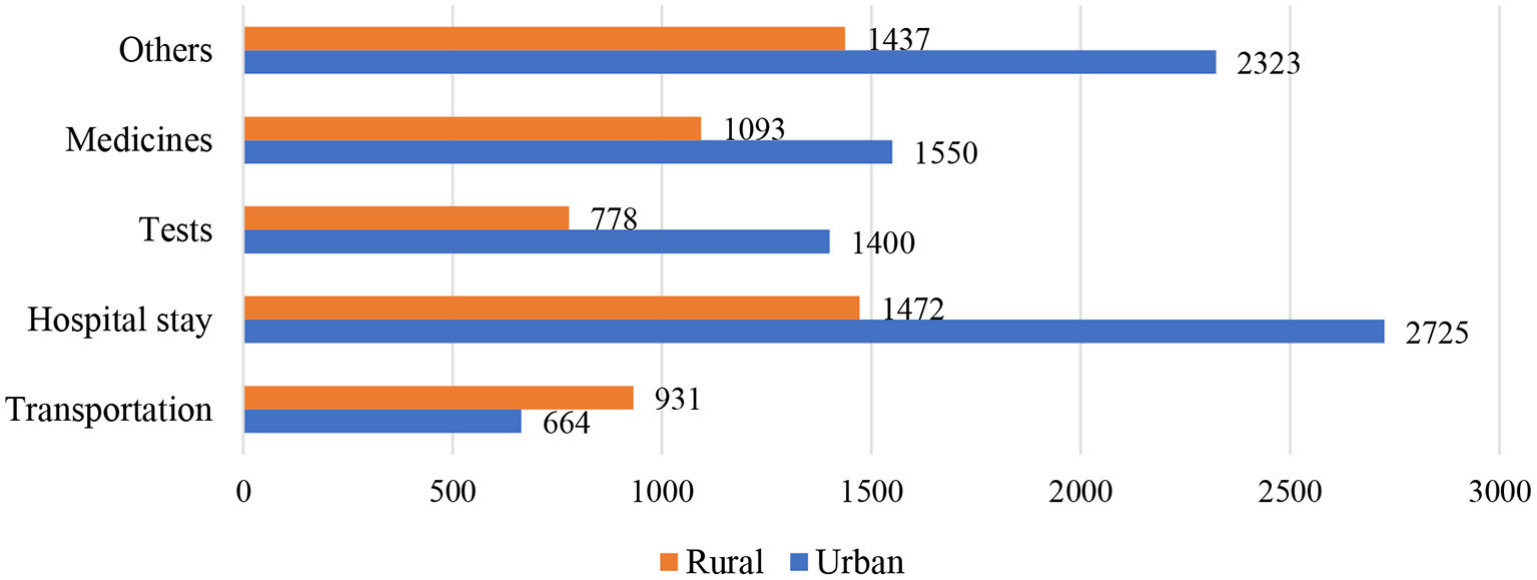
The urban–rural difference of average out-of-pocket expenditure (in rupees) of transportation, hospital stays, tests, medicines and other costs paid for delivery of the most recent live birth among women aged 15–49 years in India and selected states, 2015–2016.

Expectedly, expenditure on childbirth was higher in the private health sector than in the public health sector (Figure 5). A considerable difference in public–private expenditure was also seen nationally and across the states, Bihar and Tamil Nadu poses the least expenditure in public healthcare whereas West Bengal shows comparatively higher cost for the last birth. On the other hand, in private healthcare the cost was the highest in Tamil Nadu along with West Bengal, even more than the national level. Moreover, it can be seen that out-of-pocket costs in urban places were higher than those in a rural setting (Figure 6); therefore, it is also evident that people living in rural areas had a higher burden to access healthcare. In contrast, urban areas had better access to public and private healthcare services with a 1.5 times lower expenditure than rural counterparts (Figure 7).

An adverse socio-economic differential (Table 3) in OOPE demonstrated that people from the rural setting were more likely to spend more than urban households. Further, due to the absence of financial risk protection, high OOPE affected the poorer wealth quantile and the people from the deprived caste badly. While age (0.04), level of education (0.19), caste (0.0), religion (0.1), and wealth quantile (0.12) directly affected expenditure, husband's profession (−0.02), and place of residence (−0.0) did not show a consistent effect on OOPE.

**Table 3:** Regression coefficient and 95% CI of out-of-pocket expenditure (OOPE) (in rupees) of the most recent live birth among women aged 15–49 years according to background characteristics in India, the National Family Health Survey-4 (NFHS-4) (2015–2016).

**Background characteristics**	**Coef**.	**Std. Err**.	***t***	***P* > *t***	**(95% Conf. Interval)**
Age (15–24, 25–34, 35–49)	0.04	0.01	3.54	0.00	0.02–0.07
Place of residence (urban, rural)	−0.00	0.02	−0.00	1.00	0.0–0.04
Education (primary, secondary, higher)	0.19	0.01	20.68	0.00	0.17–0.21
Wealth quantile (poor, middle, rich)	0.12	0.01	10.67	0.00	0.10–0.14
Caste (SC, ST, non-SC/Ts)	0.00	0.01	0.28	0.78	0.0–0.02
Religion (Hindu, Muslim, Christian, others)	0.10	0.01	9.84	0.00	0.08–0.12
Husband's occupation (not working, professional or clerical, agricultural workers, services)	−0.02	0.01	−2.69	0.01	−0.04–(−0.01)
Place of delivery (public, private)	1.79	0.02	103.32	0.00	1.76–1.80
Constant	2.99	0.07	41.99	0.00	2.85

Note: *p < 0.05, **p < 0.01, ***p < 0.001.

**Figure 8:**
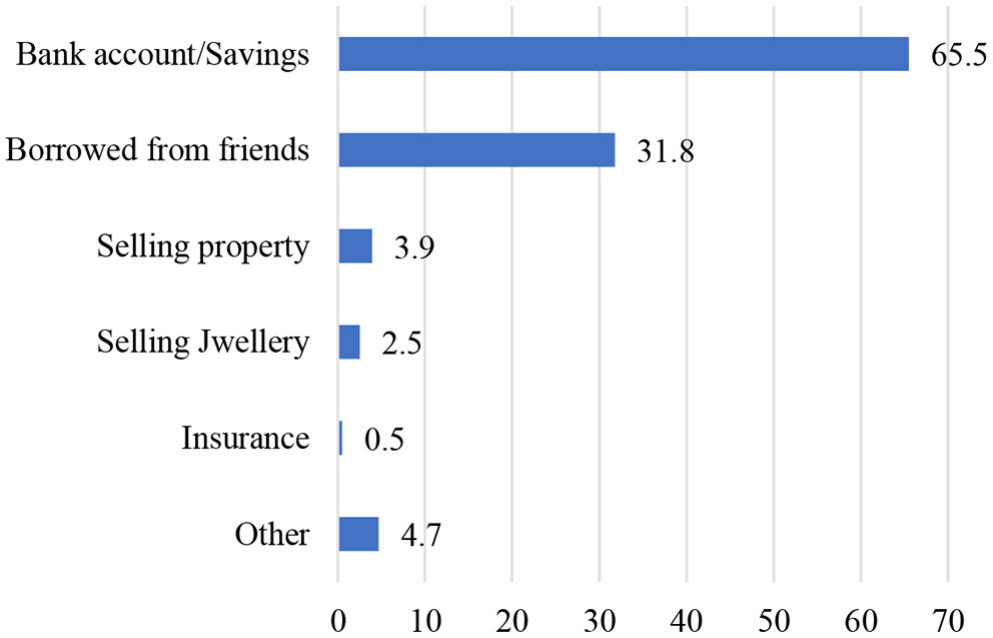
The nature of out-of-pocket cost (in rupees) paid for delivery for the most recent live birth among women aged 15–49 years in India by the poorest quantile, 2015–2016.

The analysis (Figure 8) from NFHS data showed that the high burden of the healthcare expenditure by the poorest quantile was financed primarily in two ways—by their savings (66%) and borrowing from friends or relatives (32%). In rural areas, a substantial proportion of the health expenditure for the last live birth was financed by borrowing money from others or selling property in the poorer sections. In contrast, this percentage for urban households was comparatively low. Though in India, to minimize the financial shock for the poorer section, the government launched health insurance schemes, unfortunately, a negligible proportion (0.5%) was identified.

## Discussion

The NHM in India aimed to increase the utilization and access of maternal care, reduce maternal and child deaths and institutional deliveries, and reduce the out-of-pocket expenditure on maternal care. However, evidence suggests a surge in the utilization of maternal and child care services and reduction in maternal and child deaths in the post-NHM period (22, 33–35); there are limited studies that have exploited the effectiveness of the program. This study presents a comprehensive picture of the availability, utilization, and expenditure of MCH care in the pre-NHM period.

In this study, we assessed the population-level impact of the NHM on maternal and child healthcare. Caste and economic status are the two strongest socio-economic determinants of health in the setting of India (36). Overall, we found positive impacts of the NHM on delivery care, but at the same time, a sharp socio-economic inequality persisted in the post-NHM period. Several studies also reported considerably lower beneficial impacts among the low socio-economic population groups. Besides, that caste is a factor of inequity in health has been revealed in other previous studies conducted in India. Women belonging to lower social groups (SC, ST) are less likely to be assisted by a skilled birth attendant (32). A study conducted in Kerala concluded that caste-based inequity in household health expenditure reproduces unequal access to general healthcare among different caste groups (36). A review of social exclusion, caste, and health concluded that the health outcomes and healthcare-seeking behavior of SCs and STs indicate both their social exclusion and the strong association between poverty and health for this population (37). In the examination presented in this article, we specify that caste may impact access to healthcare and the quality of care received. A substantial inter-state variation in the impacts of the NHM was found. Lower performing states such as Bihar showed a higher mortality rate, under-utilization of ANC and immunization care, and a minimal percentage of institutional births. However, states such as Tamil Nadu and West Bengal showed better MCH utilization. However, it is surprising to note that higher-performing state such as Tamil Nadu had a substantial decline in ANC and immunization care over the period (7). Moreover, conducting state-wise comprehensive evaluation studies and using quantitative and qualitative methods to identify the primary reasons for the inter-state variations in utilization of MCH care services would help to formulate practical policy guidelines.

Results confirm the growth of private healthcare (53%) across the nation's states. The National Sample Survey (NSS) stated that “not satisfied with medical treatment” ranked primarily in rural and urban areas. The reason “not satisfied with medical treatment” was followed by “lack of availability of services” in rural and “long waiting time” in urban settings. Besides, the latest NFHS data showed that the observation of “poor quality of care” was one of the most critical reasons across the states of India. A recent study suggested that the element of quality of care included interpersonal and clinical magnitudes, which stimulated the choice and utilization of healthcare (38).

The percentage of institutional deliveries doubled between 2005–2006 and 2015–2016, from 39 to 79%, and after that, interventions through the NHM contributed to a significant increase. Almost 100% of births in Tamil Nadu took place in a health facility. Rural–urban differences have also narrowed over the period (14%). A study carried out on EAG states found an enormous socio-economic inequity in the uptake of institutional delivery, preferring higher socioeconomic groups, in the pre-NHM period. A similar pattern was observed in the post-NHM period, but the magnitude of inequity in institutional delivery dropped considerably. On one hand, the JSY contributed to institutional delivery and ANC uptake. Unfortunately, states such as Bihar showed a decline in full ANC care. But, studies revealed equity and uptake of ANC improved in most states in the late post-NHM period and socio-economic inequalities also narrowed down (39).

A substantial component of total health expenditure in India comes from out-of-pocket payments. These payments include—consultation charges, investigative testing, hospital stay, medications and conveyance. The NHM was launched with the aim to cut down the out-of-pocket expenditure by providing better MCH care in the public sector. The NFHS showed that the average out-of-pocket cost for delivery for the most recent live birth was Rs. 7,938. The usual cost was five times higher in private healthcare facilities (Rs. 16,522) than the public healthcare facilities (Rs. 3,198) in the post-NHM period. The differentials in OOPE on delivery care between public and private health centers have massively enlarged; this is possibly because of the increasing incidence of cesarean deliveries across the states of India (22). One of the most significant elements of OOP expenditure is obtaining medications. The NSS (1999–2000) estimate showed that more than two-thirds of the total OOP expenditure in urban and more than three-quarters in rural areas was spent on medications. The negative socio-economic discrepancy in out-of-pocket payments was revealed because the poorest quintile in rural areas spends around 87% of OOPE on medications (40).

In contrast, the richest quintile in urban areas paid relatively less, 65% (41) for the equivalent expenditure. The high OOP payment adversely affected the deprived sections because of the absence of financial risk protection. After adjusting for health expenditure due to OOP payments, an additional 3.5% of the population fell below the poverty line, grounded on estimates for 2005–2006 (42); comparatively, a slight rising trend was observed when 3.25% fell below the poverty line from the estimate made in 1999–2000 (41, 43). Numerous observations were documented by a study of 482 poor households in Udaipur, Rajasthan, which showed that a little less than one-third of the households identified a huge health expenditure as one of the reasons for economic pressure (44). Facing this certainty, the poor often finance rising healthcare costs by cutting down consumption level expenditures for other household members (45). Thus, an ailment of a household member could have noxious consequences toward further destitution of the households (39, 41).

## Conclusion

To conclude, the NHM effectively increased the utilization continuation of maternal and child care services in public health centers and minimized expenditure on delivery care. Though the magnitude of intra-state and socio-economic inequalities has reduced, people from deprived social groups and poor developing states are still experiencing inequality. However, results also indicated that overcoming structural barriers through programs focusing on lower socio-economic groups rather than the psychological outlooks of poor people will likely to reduce inequities and indorse the uptake of maternal and child healthcare services.

## Data Availability Statement

The original contributions presented in the study are included in the article/supplementary material, further inquiries can be directed to the corresponding author.

## Ethics Statement

This analysis utilizes a secondary dataset with no identifiable information on the survey participants. This dataset is available in the public domain for study use; hence, no approval was required from any institutional review board, as there is no question of human subject protection in this case.

## Author Contributions

MB and SG conceptualized the article. MB analyzed the data and wrote the manuscript. SG edited the article. All authors approved the final version of the manuscript.

## Conflict of Interest

The authors declare that the research was conducted in the absence of any commercial or financial relationships that could be construed as a potential conflict of interest.

## Publisher's Note

All claims expressed in this article are solely those of the authors and do not necessarily represent those of their affiliated organizations, or those of the publisher, the editors and the reviewers. Any product that may be evaluated in this article, or claim that may be made by its manufacturer, is not guaranteed or endorsed by the publisher.
