# Corrigendum: Social and regional disparities in utilization of maternal and child healthcare services in india: A study of the post-national health mission period

**DOI:** 10.3389/fped.2022.980177

**Published:** 2022-07-20

**Authors:** Madhumita Bango, Soumitra Ghosh

**Affiliations:** ^1^School of Health Systems Studies, Tata Institute of Social Sciences, Mumbai, India; ^2^Centre for Health, Policy, Planning, and Management, School of Health Systems Studies, Tata Institute of Social Sciences, Mumbai, India

**Keywords:** inequalities, maternal health, child health, healthcare utilization, NHM

In the published article, there was an error in the legend for **Figure 4** “Percentage distribution of the type of healthcare facility utilized for maternal and child health (MCH) services in India, 2015–2016” on page 7. The corrected legend appears below.

**Figure 4**. Percentage distribution of utilization of healthcare facilities in India, 2015–16.

In the published article, there were typos in the text. Corrections have been made to **Results** section, Inequalities in Maternal and Child Health Expenditure Burden subsection, page 5 and page 6. The corrected sentences appears below.

“…Further, intra-state variations occurred in the utilization of healthcare services (**Figure 5**).” has been corrected to “…Further, intra-state variations occurred in the utilization of healthcare services.”

“…It is evident from the figure that there was a high dependence on the private sector for the latest delivery in West Bengal and Tamil Nadu, mainly due to the weak public delivery system” has been removed from the text.

“A considerable difference in public–private expenditure was also seen nationally and across the states, especially in the better performing states such as Tamil Nadu and West Bengal.” has been corrected to “A considerable difference in public–private expenditure was also seen nationally and across the states, Bihar and Tamil Nadu poses the least expenditure in public healthcare whereas West Bengal shows comparatively higher cost for the last birth. On the other hand, in private healthcare the cost was the highest in Tamil Nadu along with West Bengal, even more than the national level.”

A correction has been made to **Discussion** section, paragraph 2 and paragraph 3, page 7. The term “NRHM” should be changed to “NHM”. The corrected paragraphs appears below.

“In this study, we assessed the population-level impact of the NHM on maternal and child healthcare. Caste and economic status are the two strongest socio-economic determinants of health in the setting of India (36). Overall, we found positive impacts of the NHM on delivery care, but at the same time, a sharp socio-economic inequality persisted in the post-NHM period. Several studies also reported considerably lower beneficial impacts among the low socio-economic population groups. Besides, that caste is a factor of inequity in health has been revealed in other previous studies conducted in India. Women belonging to lower social groups (SC, ST) are less likely to be assisted by a skilled birth attendant (32). A study conducted in Kerala concluded that caste-based inequity in household health expenditure reproduces unequal access to general healthcare among different caste groups (36). A review of social exclusion, caste, and health concluded that the health outcomes and healthcare-seeking behavior of SCs and STs indicate both their social exclusion and the strong association between poverty and health for this population (37). In the examination presented in this article, we specify that caste may impact access to healthcare and the quality of care received.

A substantial inter-state variation in the impacts of the NHM was found. Lower performing states such as Bihar showed a higher mortality rate, under-utilization of ANC and immunization care, and a minimal percentage of institutional births. However, states such as Tamil Nadu and West Bengal showed better MCH utilization. However, it is surprising to note that higher-performing state such as Tamil Nadu had a substantial decline in ANC and immunization care over the period (7). Moreover, conducting state-wise comprehensive evaluation studies and using quantitative and qualitative methods to identify the primary reasons for the inter-state variations in utilization of MCH care services would help to formulate practical policy guidelines.”

A correction has been made to **Discussion** section, paragraph 5, page 8. The term “NRHM” should be changed to “NHM”. The corrected paragraph appears below.

“The percentage of institutional deliveries doubled between 2005–2006 and 2015–2016, from 39 to 79%, and after that, interventions through the NHM contributed to a significant increase. Almost 100% of births in Tamil Nadu took place in a health facility. Rural–urban differences have also narrowed over the period (14%). A study carried out on EAG states found an enormous socio-economic inequity in the uptake of institutional delivery, preferring higher socioeconomic groups, in the pre-NHM period. A similar pattern was observed in the post-NHM period, but the magnitude of inequity in institutional delivery dropped considerably. On one hand, the JSY contributed to institutional delivery and ANC uptake. Unfortunately, states such as Bihar showed a decline in full ANC care. But, studies revealed equity and uptake of ANC improved in most states in the late post-NHM period and socio-economic inequalities also narrowed down (39).”

The authors apologize for these errors and state that this does not change the scientific conclusions of the article in any way. The original article has been updated.

## Publisher's note

All claims expressed in this article are solely those of the authors and do not necessarily represent those of their affiliated organizations, or those of the publisher, the editors and the reviewers. Any product that may be evaluated in this article, or claim that may be made by its manufacturer, is not guaranteed or endorsed by the publisher.

